# Supercritical CO_2_-Derived Tomato Extract Activates Signaling Pathways to Reduce Oxidative Stress and Inflammation in Astrocyte Cells

**DOI:** 10.3390/nu18091464

**Published:** 2026-05-03

**Authors:** Serena Recalchi, Beatrice Mengoni, Barbara Scaglia, Marilena Esposito, Emiliano Montalesi, Valeria Manganelli, Gloria Riitano, Elena Fasciolo, Tuba Rana Caglar, Daniela Caissutti, Camilla Moliterni, Federica Armeli, Rita Businaro, Roberta Misasi, Maurizio Sorice, Antonella Capozzi

**Affiliations:** 1 Department of Experimental Medicine, Sapienza University of Rome, 00100 Rome, Italy; serena.recalchi@uniroma1.it (S.R.); valeria.manganelli@uniroma1.it (V.M.); gloria.riitano@uniroma1.it (G.R.); elena.fasciolo@uniroma1.it (E.F.); tubarana.caglar@uniroma1.it (T.R.C.); daniela.caissutti@uniroma1.it (D.C.); c.moliterni@uniroma1.it (C.M.); maurizio.sorice@uniroma1.it (M.S.); antonella.capozzi@uniroma1.it (A.C.); 2 Department of Life, Health and Health Professions Sciences, Link Campus University, 00100 Rome, Italy; 3 Department of Medico-Surgical Sciences and Biotechnologies, Sapienza University of Rome, 00100 Rome, Italy; beatrice.mengoni@uniroma1.it (B.M.); federica.armeli@uniroma1.it (F.A.); rita.businaro@uniroma1.it (R.B.); 4 Department of Agricultural and Environmental Sciences Production, Landscape, Agroenergy (DiSAA), University of Milan, 20100 Milan, Italy; barbara.scaglia@unimi.it (B.S.); marilena.esposito@unimi.it (M.E.); 5 Department of Oncology and Molecular Medicine, ISS Rome, 00161 Rome, Italy; emiliano.montalesi@iss.it

**Keywords:** supercritical CO_2_ extraction, tomato extracts, oxidative stress, nutraceutical

## Abstract

Background/Objectives: In this study, we investigated the effect on antioxidant defenses of a tomato extract obtained by supercritical CO_2_ extraction (sCO_2_TE), evaluating whether this green extraction method preserves biological activity compared to a conventional tomato extract (CTE) and focusing on superoxide dismutase (SOD) and glutathione peroxidase (GPx) regulation, Nuclear factor erythroid 2-related factor 2 (NRF2) activation, reactive oxygen species (ROS) and lipid peroxidation modulation. Methods: Human glioblastoma astrocytoma U-373 cells were pre-treated with sCO_2_TE or conventional tomato extract (CTE) and subsequently exposed to sodium arsenite (AsNaO_2_) to induce oxidative stress, or lipopolysaccharide (LPS) to trigger inflammatory signaling. Cell viability was assessed by Trypan Blue and MTT [3-(4,5-dimethylthiazolyl-2)-2,5-diphenyltetrazolium bromide]; cell toxicity by propidium iodide staining. Intracellular ROS and lipid peroxidation were measured by flow cytometry. Gene expression of NRF2, SOD1 and GPX1 was analyzed by qRT-PCR, NRF2 activation and modulation of ERK1/2 (Extracellular Signal-Regulated Kinase 1/2) and NF-κB (Nuclear Factor kappa-light-chain-enhancer of activated B cells) were evaluated by Western blot. Results: Pre-treatment with sCO_2_TE significantly reduced AsNaO_2_-induced ROS production and lipid peroxidation, showing a stronger effect compared to CTE. sCO_2_TE enhanced the expression of NRF2 phosphorylation and its downstream targets SOD1 and GPX1, particularly under oxidative stress conditions. In addition, sCO_2_TE attenuated LPS-induced phosphorylation of ERK1/2 and NF-κB p65, suggesting anti-inflammatory activity. Conclusions: These findings demonstrate that sCO_2_TE preserves the antioxidant and anti-inflammatory properties of tomato-derived bioactives. The comparable efficacy of sCO_2_TE and CTE supports the use of sCO_2_ as a sustainable and solvent-free extraction method for the development of nutraceutical formulations targeting oxidative stress and neuroinflammation.

## 1. Introduction

Oxidative stress, characterized by an imbalance between the production of free radicals, such as ROS and RNS, and the body’s antioxidant defenses, leads to the release of factors that stimulate the cells of the immune system to produce pro-inflammatory mediators that amplify the damage at a systemic level and constitute one of the main risk factors for the development of chronic-degenerative diseases [1]. Oxidative stress and inflammation are biunivocally linked since excess of ROS. Excess ROS can further amplify inflammatory signaling, and inflammatory mediators enhance ROS production, creating a self-perpetuating cycle that drives cellular dysfunction, tissue damage and disease progression [2,3,4].

The brain is highly susceptible to oxidative stress due to the abundance of peroxidable polyunsaturated fatty acids at the level of the myelin sheaths, the high content of iron—which is an endogenous catalyzer for the generation of reactive species—and the scarcity of antioxidant enzymes, compared to other organs [5]. This susceptibility is amplified in elderly people and even more enhanced in those with neurodegenerative conditions [6].

Cells have evolved antioxidant defense, including the transcription factor NRF2, a central regulator to maintain redox homeostasis involved in the regulation of more than 200 cytoprotective genes, such as SOD and GPX [7,8]. This antioxidant system helps to neutralize ROS, thereby contributing to redox balance and cell survival.

Astrocytes, the most abundant glial cells in the central nervous system, play essential roles in maintaining neuronal health, including trophic support, regulation of extracellular ion and neurotransmitter balance, maintenance of the blood–brain barrier and scavenging of ROS [9].

In this context, neuroprotection emerges as a central therapeutic strategy aimed at preserving neuronal integrity and function. Many attempts are currently being made in order to delay the progression of Alzheimer’s disease by reducing inflammatory mechanisms underlying the disease. Several studies support a relationship among neuroinflammation and nutrients, foods or dietary patterns, including natural antioxidant and anti-inflammatory compounds found in plant foods such as fruits, particularly berries [10,11,12,13]. Approaches that enhance antioxidant defenses, restore mitochondrial homeostasis or modulate glial reactivity have shown promise in experimental models. Many plants contain polyphenolic antioxidants and anti-inflammatory factors; indeed, nutraceuticals, phytochemicals and other bioactive compounds are increasingly investigated for their potential to attenuate oxidative stress and neuroinflammation. Several studies were conducted using in vitro cell lines or in vivo models including zebrafish, rats and various transgenic or toxin-induced mice [14,15,16,17].

Given the role of tomato-derived nutraceuticals to modulate oxidative stress pathways, a nutraceutical product rich in lycopene (LYC) was shown to increase the astrocyte antioxidant activity in Alzheimer’s disease models [18,19]. In particular, the use of LYC in animal models has demonstrated effectiveness in inhibiting inflammation as well as oxidative stress [16,20].

Tomato pomace (TP) is the residue of the production of tomato sauce, and it is considered an interesting alternative as LYC feedstock in the perspective to enhance LYC bioavailability. During the process, the tomatoes were cooked, and the peel and seed were separated from the pulp. Cooking causes a partial isomerization of the native LYC, thus higher *Z* fractions, mainly extracted from the seed. Previous data reported a ratio between peel and seed extraction of 1:10; thus, the seed component greatly affects the final bioactive characteristics, including bioavailability [21]. The most evident is the nature of the extract that is a tomato oliolite. Since oil in seed accounted for 30% of dry matter and a different composition in bioactive molecules, and to avoid the use of organic solvents, the scientific literature widely explored supercritical CO_2_ extraction (sCO_2_) as alternative technology [22,23,24,25]. sCO_2_ enables the recovery of heat- and oxidation-sensitive compounds, such as polyunsaturated fatty acids (ω-3, ω-6), vitamins, cannabinoids, flavonoids, sterols, tocopherols and other high value-added molecules, while preserving their structural and functional integrity. The resulting extracts are characterized by exceptional purity and highly refined organoleptic properties. Taking into consideration the mass balance of the process, Scaglia et al. highlighted that no LYC degradation occurred, as well as the isomerization of LYC [25]. The optimization of supercritical extraction conditions (temperature, pressure and time) gives high recovery of LYC; however, the great solubility of *Z* fractions in the oil enhanced its recovery together with improved quality being solvent-free.

In this study, we propose to investigate the ability of TE, obtained by sCO_2_, to upregulate antioxidant enzymes (SOD, GPx), activate NRF2 signaling and reduce both ROS release and lipid peroxidation in glioblastoma astrocytoma cells after oxidative stress induction.

## 2. Materials and Methods

### 2.1. Extraction of Tomato Pomace

TP extract, obtained by supercritical CO_2_ extraction of TP (sCO_2_TE), was achieved from TP samples, using full-scale tomato cannery pomace [21]. TP was partially dried under vacuum at 40 °C to reach a residual moisture content of 15–18% wet weight (*w*/*w*) and grinded to obtain powder (≤0.1 mm) using a ball mill (Retsch MM200, 2.5 min, 30 Hz; Retsch GmbH, Haan, Germany). The sample was then extracted using a supercritical CO_2_ extractor (Separeco S.r.l., Pinerolo Turin, Italy) [21]. Briefly, around 1 kg of TP was extracted at pressure of 380 bar CO_2_, flow of 15 kg/h and temperature of 80 °C. Sample was stored in glass vacuum-sealed box in the freezer at −22 °C.

A reference sample of tomato extracted conventionally (CTE) is currently available for purchase from Sigma Aldrich (Sigma Aldrich, Milan, Italy). The CTE was stored at −22 °C in the original packing.

### 2.2. Analytical Characterization of the Extracts

LYC quantification was performed on an Agilent 1260 Infinity HPLC system (Agilent Technologies, Inc., Santa Clara, CA, USA) equipped with a C30 Develosil^®^ RPAQUEOUS column (5 µm, 250 × 4.6 mm).

Twenty µL of CTE and sCO_2_TE extract, diluted in methanol to obtain a final concentration of 1.6 mg/mL and 50 mg/mL, respectively, were injected at 10 °C with a mobile phase composed of a mixture of methyl tert-butyl ether (A) and methanol (B), and the following compositional gradient was applied as per reference [25] with some slight modifications: from 0 min to 70 min 15% (A) and 85% (B); 5 min 55% (A) and 45% (B); 75 min 95% (A) and 5% (B). Flow rate was set at 1.3 mL min^−1^. The LYC was detected with an Agilent UV/Vis spectrophotometer at 475 nm, and the peaks were attributed to the different isomers based on the attribution proposed in the literature [21,22,23,24,25,26,27]. The sCO_2_TE was examined for fatty acid, tocopherol and phytosterol content.

Analytic characterization of fatty acids, tocopherols and phytosterols was performed according to a specific procedure [28,29] extensively described in the Supplementary Materials.

### 2.3. Cell Cultures and Treatments

Human glioblastoma astrocytoma cells U-373 MG (American Type Culture Collection, ATCC, Manassas, VA, USA) were grown in DMEM F-12 medium (Sigma-Aldrich, Milan, Italy) supplemented with 10% fetal bovine serum (FBS, Aurogene S.r.l., Rome, Italy), 100 units/mL penicillin and 10 mg/mL streptomycin (Aurogene S.r.l.) and maintained in a humified atmosphere of 37 °C and 5% CO_2_.

The sCO_2_TE was solubilized in Dimethyl Sulfoxide (DMSO, Sigma-Aldrich) and CTE, used as control, was solubilized in Tetrahydrofuran (THF, Sigma-Aldrich). The commercial CTE produces a more complex mixture containing hydrophobic components requiring THF for effective solubilization.

In all the experiments, there is a sample of untreated cells, incubated only with culture medium plus DMSO or THF in the same quantity used to solubilize the extract used, defined by convention as the vehicle. Moreover, cells were exposed to sodium arsenite (AsNaO_2_) to induce oxidative stress [29] or to lipopolysaccharide (LPS) to activate inflammatory signaling pathways [30].

### 2.4. Cell Viability Assay

U-373 cells, following treatment, were assessed by Trypan Blue (TB, Sigma-Aldrich) and MTT [3-(4,5-dimethylthiazolyl-2)-2,5-diphenyltetrazolium bromide, ATCC] assays to investigate proliferation and viability.

For the TB assay, cells were seeded into a 12-well plate at a density of 2.5 × 10^5^ cells/mL per well, pre-treated with increasing concentrations of sCO_2_TE or CTE (10 μg/mL; 50 μg/mL; 100 μg/mL; 200 μg/mL; 500 μg/mL) for 1 h, stimulated with an increasing concentration of ArNaO_2_ (10 μM; 50 μM; 100 μM; 200 μM; 500 μM) for 2 h and then analyzed by TB assay. The MTT assay was performed according to the manufacturer’s instructions. In brief, cells were plated in a 96-well plate at a density of 8 × 10^3^ cells. After treatments, 10 μL of MTT (5 mg/mL) were added to each well, and those containing only medium were considered blank wells. The reaction was allowed to proceed for 4 h at 37 °C. The culture medium was then removed, and the formed formazan crystals were dissolved by adding 200 μL of DMSO. The absorbance at 570 nm of each well was then read on a GLOMAX plate reader (Promega Corporation, Madison, WI, USA) and was directly related to the number of viable cells. All the samples and related measurements were carried out in triplicate.

### 2.5. Propidium Iodide (PI) Staining

For PI staining, U-373 cells were plated in 12-well culture plates at a density of 2.5 × 10^5^ cells/mL per well. Following pre-treatment for 1 h with both tomato extracts (sCO_2_TE or CTE, 100 μg/mL) and incubation with ArNaO_2_ (200 μM) for 2 h, cells were harvested and separated from the culture medium by centrifugation. The pellets were then washed once with Phosphate-Buffered Saline (PBS, Aurogene S.r.l.) and subsequently fixed in 70% ethanol in PBS for 1 h at 4 °C. After fixation, cells were washed twice with PBS, resuspended in 125 μL of PBS containing 12.5 μL of RNase (5 μg/mL, Sigma-Aldrich) and stained with 125 μL of PI (100 μg/mL, Sigma-Aldrich). Samples were incubated for 30 min at room temperature (RT) in the dark and then used to analyze DNA content. Fluorescence was recorded using a CytoFLEX cytometer (Beckman Coulter, Brea, CA, USA).

### 2.6. Flow Cytometric Analysis of Reactive Oxygen Species (ROS) Production

The intracellular production of ROS was detected using 2′,7′-dichlorofuorescin diacetate (DCFDA) through a cellular ROS Detection Assay Kit (Abcam, Cambridge, UK), following the manufacturer’s instructions. In brief, U-373 cells were pre-treated with both tomato extracts (sCO_2_TE or CTE, 100 μg/mL) for 1 h and stimulated with sodium AsNaO_2_ (200 μM) for 2 h, the culture medium was removed, and cells were washed twice in PBS. Then, 500 μL/well DCFH-DA (1 μM) was added and incubated at 37 °C for 15 min, then samples were washed three times with PBS. Furthermore, 0.15% trypsin digestion solution was added, and cells were collected and centrifuged. Finally, fluorescence was measured by flow cytometry using CytoFLEX (Beckman Coulter). Background signals were subtracted from raw fluorescence values, and the results were expressed as mean fluorescence intensity (MFI). Each experimental condition was tested in three independent replicates.

### 2.7. Lipid Peroxidation Assay

The stock solution of C11-BODIPY^581/591^ undecanoic acid (Lipid Peroxidation Sensor, Life technologies-Invitrogen, Carlsbad, CA, USA) was prepared by dissolution in DMSO to have the concentration of 10 mM.

Following pre-treatment with sCO_2_TE or CTE (100 μg/mL) for 1 h and stimulation with sodium AsNaO_2_ (200 μM) for 2 h, U-373 cells were incubated for 30 min at 37 °C with C11-BODIPY^581/591^ at the final concentration of 10 μM in growth medium. After incubation the cells were washed three times with PBS and FITC, fluorescence was measured by flow cytometry using CytoFLEX (Beckman Coulter). Background signals were subtracted from raw fluorescence values, and the results were expressed as MFI relative to the vehicle. Each experimental condition was tested in three independent replicates.

### 2.8. Real-Time qPCR of SOD-1, GPX and NRF2

U-373 cells were seeded in 6-well plates at a density of 1 × 10^6^ cells/mL per well. After pre-treatment with sCO_2_TE or CTE (100 μg/mL) for 1 h, the oxidative stimulus, AsNaO_2_ (200 μM) was added for 2 h. After treatments, cells were lysed with 700 μL of Qiazol Lysis Reagent (Qiagen, Hilden, Germany) and stored at −80 °C. Total RNA was extracted using the miRNeasy Micro kit (Qiagen, Hilden, Germany) and quantified by NanoDrop One/OneC (Thermo Fisher Scientific, Waltham, MA, USA). The cDNA was synthesized using the high-throughput reverse transcription kit. Quantitative real-time PCR (qPCR) was performed for each sample in triplicate on an Applied Biosystems 7900HT fast real-time PCR system (Applied Biosystem, Cheshire, UK), using Power SYBR^®^ Green PCR Master Mix. Primers for real-time PCR amplification were designed through UCSC GENOME BROWSER https://genome.cse.ucsc.edu/ (accessed on 1 November 2022; University of California, Santa Cruz, CA, USA) (Table 1). Analysis of real-time PCR data was performed using the comparative threshold cycle (CT) method. The target quantity, normalized against the endogenous GAPDH reference primer (ΔCT) and against the untreated control calibrator (ΔΔCT), was calculated by the 2^−ΔΔCT^ equation.

### 2.9. Evaluation of Phospho-NRF2 by Western Blot Analysis in U-373 Cells

For Western blot, 5 × 10^5^ of U-373 cells were plated and treated as previously described. After treatment, the medium was removed, and the cells were washed once in PBS and harvested by trypsinization. Nuclear extracts were prepared as previously described [31]. Briefly, cells were resuspended in buffer A (20 mM HEPES, pH 7.2; 0.1% Nonidet P-40, 20 mM KCl, 3.0 mM MgCl2, 1 mM Na_3_VO_4_, 5 mM DTT and protease inhibitors cocktail), after 30 min on ice, and then centrifuged for 30 min at 10,000× *g* at 4 °C. Pellets were resuspended in buffer B (40 mM HEPES, pH 7.2; 0.84 M NaCl, 0.4 mM EDTA, 50% glycerol, 1 mM Na_3_VO_4_, 5 mM DTT and protease inhibitors cocktail), after 1 h on ice, samples were centrifuged at 10,000× *g* for 1 h at 4 °C and supernatants (nuclear extracts) were transferred to new vials. The total protein concentration in each sample was determined by Bradford assay (Bio-Rad, Segrate, MI, Italy).

The equal nuclear fraction proteins were blotted onto polyvinylidene difluoride (PVDF) membranes (Bio- Rad, Segrate, MI, Italy). Membranes were blocked with 5% defatted dried milk in Tris-buffered saline, containing 0.05% Tween-20 (T-TBS) and then incubated with rabbit anti phospho-NRF2 monoclonal antibody (mAb) (Cell Signaling Technology, Danvers, MA, USA) and after washing with horseradish peroxidase-conjugated anti-rabbit IgG Ab (Sigma-Aldrich).

The anti-phospho-NRF2 Ab was stripped, and the PVDF membrane was reprobed with polyclonal anti-HISTONE H1 Ab (Abcam), as a control for loading. Immunoreactivity was assessed by the chemiluminescence reaction, using the Clarity Western ECL substrate detection system (Bio-Rad, Segrate, MI, Italy). Densitometric analysis was performed by MacBook Pro M1 (Apple Computer International, Cupertino, CA, USA), using National Institutes of Health (NIH) ImageJ 1.62 software. The density of each band (absolute value) in the same gel was analyzed.

### 2.10. Western Blot Analysis ERK1/2 and p65-NF-κB Proteins in U-373 Cells

U-373 cells were pre-treated 1 h with both tomato extract (100 μg/mL) and stimulated 1 h with LPS (100 ng/mL), then collected as described above. To prepare whole-cell extracts, cells were resuspended in lysis buffer, containing 20 mM HEPES, pH 7.2; 1% Nonidet P-40, 10% glycerol, 50 mM NaF, 1 mM Na_3_VO_4_ and protease inhibitors cocktail (Sigma-Aldrich). Soluble proteins were recovered after centrifugation of lysates at 15,000 × *g* for 15 min at 4 °C. The nuclear extracts were prepared as described above. The total protein concentration in each sample was determined by Bradford assay (Bio-Rad, Segrate, MI, Italy). After SDS-PAGE, the equal whole and nuclear fraction proteins were transferred onto PVDF membranes (Bio-Rad), and after blocking (as above), were incubated overnight with rabbit anti-phospho-ERK1/2 Ab (Cell Signaling Technology) and rabbit anti-phospho-NF-κB-p65 (Cell Signaling Technology), respectively. After washing, this reaction was followed by incubation with HRP-conjugated anti-rabbit IgG antibody (Sigma-Aldrich). As a control for loading of preparation, membranes were stripped and reprobed with rabbit anti-total ERK1/2 (Cell Signaling Technology) or polyclonal anti-HISTONE H1 Ab (Abcam). Immunoreactivity was detected using the ECL Western blotting detection system. Densitometric analysis was performed by MacBook Pro M1 (Apple Computer International, Cupertino, CA, USA), using National Institutes of Health ImageJ 1.62 software. The density of each band (absolute value) in the same gel was analyzed.

### 2.11. Statistical Analysis

The Shapiro–Wilk test was applied to test data normality. Normally distributed data were analyzed using an exploratory one-way-ANOVA; post hoc tests with appropriate multiple comparison correction were used if the ANOVA was significant. A *p*-value less than 0.05 was considered significant. Statistical analysis was performed using the Prism version 7 (GraphPad Software, San Diego, CA, USA).

## 3. Results

### 3.1. sCO_2_TE Characterization

**Figure 1:**
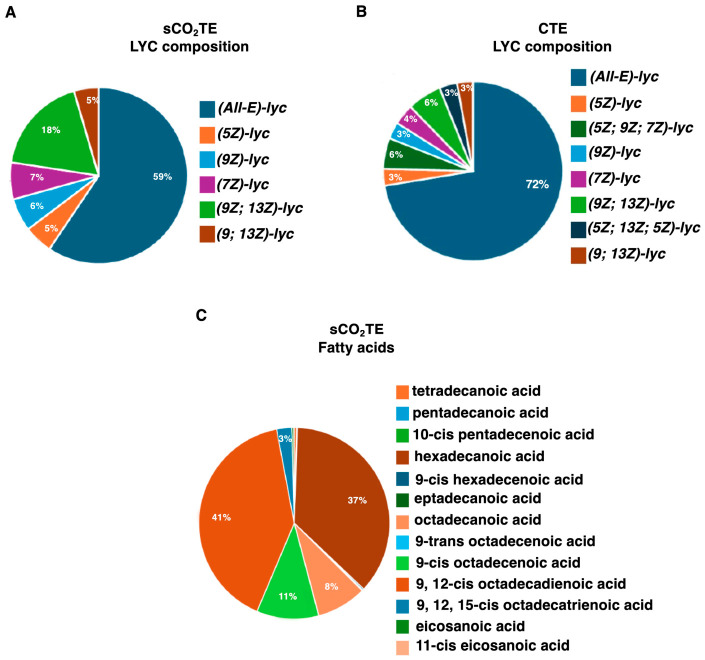
Characterization of supercritical CO_2_ tomato extract (sCO_2_TE). Lycopene isomer composition of the supercritical CO_2_ tomato extract (sCO_2_TE) (**A**) and conventional tomato extract (CTE) (**B**). Fatty acid composition of sCO_2_TE (**C**).

The LYC content of the TP obtained by supercritical CO_2_ extraction (sCO_2_TE) was 1.34 mg g^−1^ (Table 2). Biochemical analysis revealed that in sCO_2_TE, due to the different extraction characteristics and the isomerization that occurred during heating of the tomato, the all-E content was quite limited, leaving 41% of the Z form, with greater bioavailability [32] (Figure 1A). On the contrary, in CTE, all-E represented 72% of the LYC, consistent with the range reported for raw tomato fruit (Figure 1B). Other bioactives are shown in Table 2. Moreover, in sCO_2_TE, the presence of significant seed fractions completely changed the chemical composition of the oliolite, rather than the oleoresin. First, the oil presents an interesting composition in terms of fatty acids (Figure 1C). Linoleic acid is the main form which, together with other mono- and polyunsaturated molecules, is potentially interesting from a medical point of view. Both extracts present a significant content of tocopherols; in the case of sCO_2_TE, the α and γ forms were detected [33]. Finally, sCO_2_TE presents phytosterols with the most relevant molecules Stigmasterol and β-Sitosterol [33,34].

### 3.2. Cytotoxic, Anti-Proliferative and Pro-Apoptotic Effect of sCO_2_TE Extract

**Figure 2:**
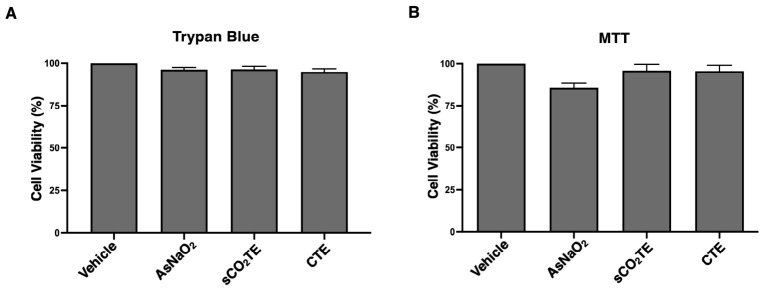
Effect of supercritical CO_2_ tomato extract (sCO_2_TE) on cell viability. Human glioblastoma astrocyte cells U-373 were treated with sCO_2_TE (100 μg/mL), conventional tomato extract (CTE, 100 μg/mL) or sodium arsenite (AsNaO_2_, 200 μM). The number of viable cells was determined by Trypan Blue (TB) exclusion test. Data are reported as the mean ± standard deviation (SD) among 10 independent experiments (**A**). Cell proliferation was measured by 3-(4,5-dimethylthylthiazol-2-yl)-2,5-diphenyltetrazolium bromide (MTT) assay. Data are reported as the mean ± standard deviation (SD) among 5 independent experiments performed in triplicate and represent cell viability as a percentage of vehicle (**B**).

In U-373 cells, TB exclusion assay revealed minimal cell viability reduction after treatment with sCO_2_TE, comparable to the CTE. Moreover, also AsNaO_2_ induced a non-significant reduction in cell viability (Figure 2A). These results were further supported by the MTT assay, which showed complete preservation of mitochondrial metabolic activity following exposure to the tomato extract as well as AsNaO_2_ (Figure 2B). Therefore, the results obtained from this preliminary study, performed in U-373 cells, excluded the cytotoxic and anti-proliferative effect of the molecules used in our experimental conditions (concentration range 10–500 μg/mL for tomato extracts and 10–500 μM for AsNaO_2_) (Figure S1).

**Figure 3:**
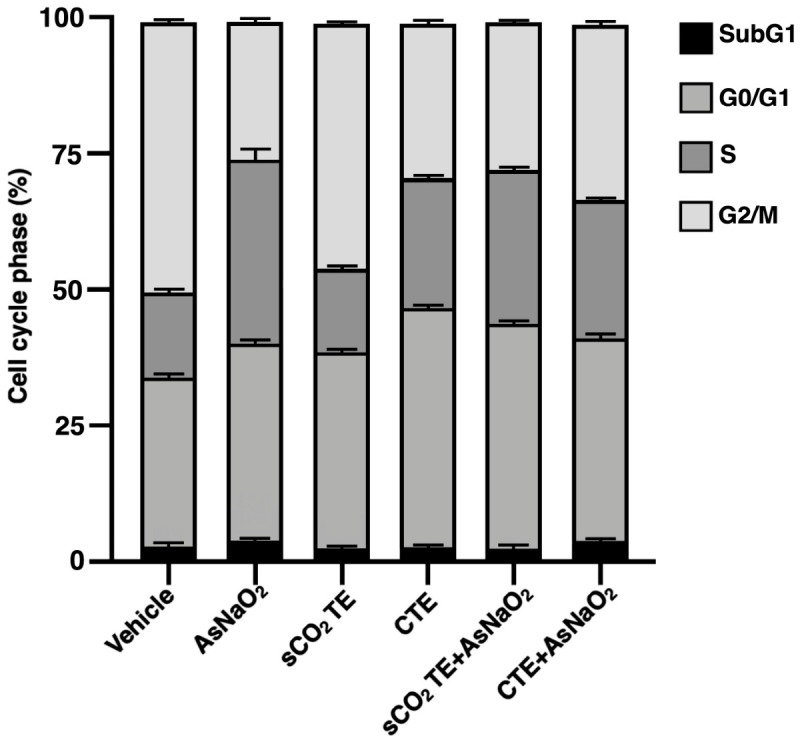
Effect of supercritical CO_2_ tomato extract (sCO_2_TE) on DNA. Human glioblastoma astrocyte cells U-373 were treated with sCO_2_TE (100 μg/mL), conventional tomato extract (CTE, 100 μg/mL) or sodium arsenite (AsNaO_2_, 200 μM) and, after staining with propidium iodide (PI), were analyzed by flow cytometric analysis to evaluate cell cycle phases. Data are reported as the mean ± standard deviation (SD) among 3 independent experiments.

Propidium iodide staining was used to quantify the sub-G0/G1 population, representing cells with reduced DNA content, typically associated with DNA fragmentation and cell death. The percentage of U-373 cells in the sub-G0/G1 fraction remained low following treatment with sCO_2_TE, CTE or AsNaO_2_, indicating no significant increase in cell death under these conditions (Figure 3) [35].

### 3.3. sCO_2_TE Modulates Oxidative Stress and Lipid Peroxidation

In order to evaluate, in U-373 cells, the antioxidant potential activity of sCO_2_TE, intracellular ROS levels were measured by flow cytometry using the cell-permeable probe DCFH-DA, which is oxidized by ROS to the highly fluorescent compound DCF.

**Figure 4:**
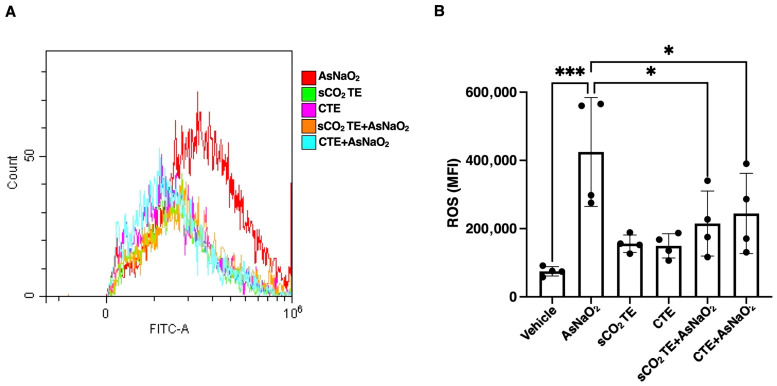
Analysis of intracellular reactive oxygen species (ROS) after supercritical CO_2_ tomato extract (sCO_2_TE) treatment. Human glioblastoma astrocyte cells U-373 were treated with sCO_2_TE (100 μg/mL), conventional tomato extract (CTE, 100 μg/mL) or sodium arsenite (AsNaO_2_, 200 μM), and alternatively they were pre-treated with sCO_2_TE (100 μg/mL) and then stimulated with AsNaO_2_ (200 μM). After treatments, ROS production was assessed using the DCFH-DA fluorescent probe. Flow cytometric histograms show measurement of ROS levels through flow cytometry analysis (**A**). Quantified ROS levels: histograms show the mean fluorescence intensity (MFI) (**B**). The results are presented as mean ± SD from 3 independent experiments. Statistical analysis indicates: * *p* < 0.05 and *** *p* < 0.001.

As shown in Figure 4A,B, AsNaO_2_ exposure markedly increased DCF fluorescence, indicating elevated ROS production. However, pre-treatment with sCO_2_TE significantly reduced DCF fluorescence intensity, modulating the AsNaO_2_ effect. These results suggest a protective antioxidant effect. Notably, the reduction in ROS levels was more evident in cells pre-treated with sCO_2_TE extract compared to those treated with the CTE.

**Figure 5:**
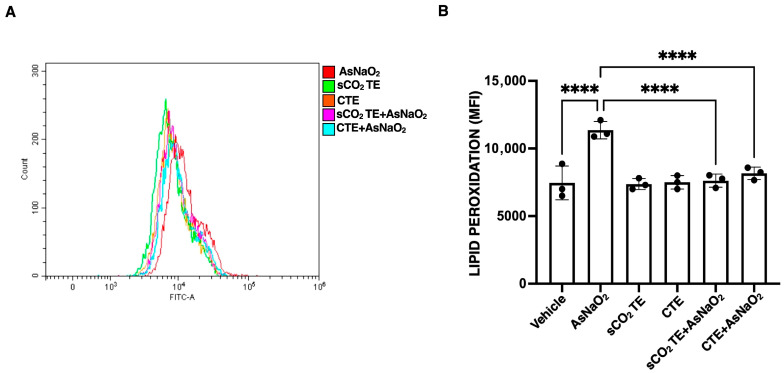
Analysis of lipid peroxidation after supercritical CO_2_ tomato extract (sCO_2_TE) treatment. Human glioblastoma astrocyte cells U-373 were treated with sCO_2_TE (100 μg/mL), conventional tomato extract (CTE, 100 μg/mL) or sodium arsenite (AsNaO_2_, 200 μM), and alternatively they were pre-treated with sCO_2_TE (100 μg/mL) and then stimulated with AsNaO_2_ (200 μM). After treatments, lipid peroxidation was evaluated using BODIPY 581/591 C11probe (Lipid Peroxidation Sensor that results in a FITC emission). Flow cytometric histograms show measurement of lipid peroxidation through flow cytometry analysis (**A**). Quantified lipid peroxidation, histograms show the mean fluorescence intensity (MFI) (**B**). The results are presented as mean ± SD from 3 independent experiments. Statistical analysis indicates: **** *p* < 0.0001.

To investigate whether sCO_2_TE might mitigate lipid peroxidation induced by oxidative stress, U-373 cells, pre-treated with sCO_2_TE and stimulated with AsNaO_2_, were incubated with the fluorescent probe BODIPY™ 581/591 C11, which incorporates into lipid bilayers and results in a FITC emission upon oxidation. This allowed the monitoring of oxidative alterations induced by AsNaO_2_ and the potential protective effects of the tomato extract. As expected, AsNaO_2_ treatment leads to a significant increase in green fluorescence, as shown in Figure 5A, reflecting increased membrane lipid peroxidation. Pre-treatment with sCO_2_TE, as well as with CTE used as a control, significantly reduced fluorescence, suggesting a protective effect against AsNaO_2_-induced lipid peroxidation (Figure 5B). These findings are consistent with the antioxidant properties of sCO_2_TE and support its potential in preventing membrane oxidative damage.

### 3.4. sCO_2_TE Enhances NRF2, SOD1 and GPX mRNA Levels

**Figure 6:**
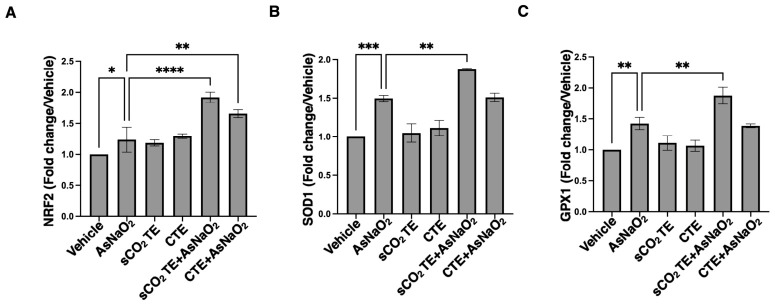
Analysis of NRF2, SOD1 and GPX after supercritical CO_2_ tomato extract (sCO_2_TE) treatment. mRNA expression of NRF2 (**A**), SOD1 (**B**) and GPX (**C**) was evaluated by qRT-PCR. Data are shown as mean ± SD from 3 independent experiments performed in triplicate. Expression profiles were determined using the 2^−ΔΔCT^ method. Statistical analysis indicates: * *p* < 0.05; ** *p* < 0.01; *** *p* < 0.001, **** *p* < 0.0001.

As shown in Figure 6, in U-373 cells, AsNaO_2_ treatment induced significant increases in NRF2 (Figure 6A), SOD1 (Figure 6B) and GPX1 (Figure 6C) expression, demonstrating the well-established role of arsenic in generating oxidative stress with a consequent activation of antioxidant response. Tomato extracts elicited a modest increase in mRNA levels of these molecules; nevertheless, in combination with AsNaO_2_, the response was significantly greater, suggesting that bioactive antioxidant compounds present in the extract (e.g., lycopene) may potentiate the activation of antioxidant signaling.

This synergistic effect likely reflects the combined action of AsNaO_2_-mediated oxidative stress and the antioxidant capacity of tomato extracts, with a better response for the one extracted using supercritical CO_2_. These results support the idea that this extraction method better preserves the antioxidant properties of tomato bioactives.

### 3.5. sCO_2_TE Induces NRF2 Activation

Since the fundamental role played by NRF2 in controlling the expression of antioxidant genes that ultimately exert anti-inflammatory functions, we studied the level of activation of this transcription factor in U-373 cells exposed to sCO_2_TE and CTE, used as control.

**Figure 7:**
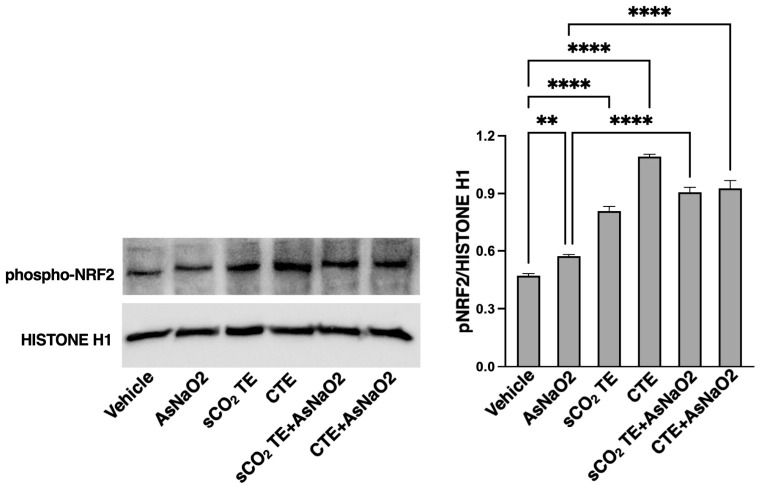
Analysis of NRF-2 phosphorylation after supercritical CO_2_ tomato extract (sCO_2_TE) treatment. Human glioblastoma astrocyte cells U-373 were treated with sCO_2_TE (100 μg/mL), conventional tomato extract (CTE, 100 μg/mL) or sodium arsenite (AsNaO_2_, 200 μM), and alternatively they were pre-treated with sCO_2_TE (100 μg/mL) and then stimulated with AsNaO_2_ (200 μM). After treatments, cells were lysed and analyzed by Western blot. Nuclear cell extracts were analyzed to verify phosphorylated NRF2 using rabbit anti-phospho-NRF2 Ab. As a control, for loading and purity of preparation, membrane was stripped and reprobed with polyclonal anti-HISTONE H1 Ab. Densitometric phospho-NRF2/HISTONE H1 ratios are shown in the right panel. Results represent the mean ± SD from 3 independent experiments. Statistical analysis indicates: ** *p* < 0.01; **** *p* < 0.0001.

Western blot analysis demonstrates that sCO_2_TE, as well as CTE, significantly upregulated phosphorylated NRF2 (Figure 7), which represents the active nuclear isoform of the protein, whose increased expression usually correlates with excessive ROS production. Moreover, pre-treatment with both tomato extracts increases NRF2 phosphorylation in samples stimulated with AsNaO_2_. These findings suggest that the oxidative environment induced by AsNaO_2_ may be attenuated by sCO_2_TE and underscore their potential in modulating oxidative stress pathways and enhancing cellular antioxidant defenses.

### 3.6. sCO_2_TE Modulates ERK1/2 and NF-kB Phosphorylation

**Figure 8:**
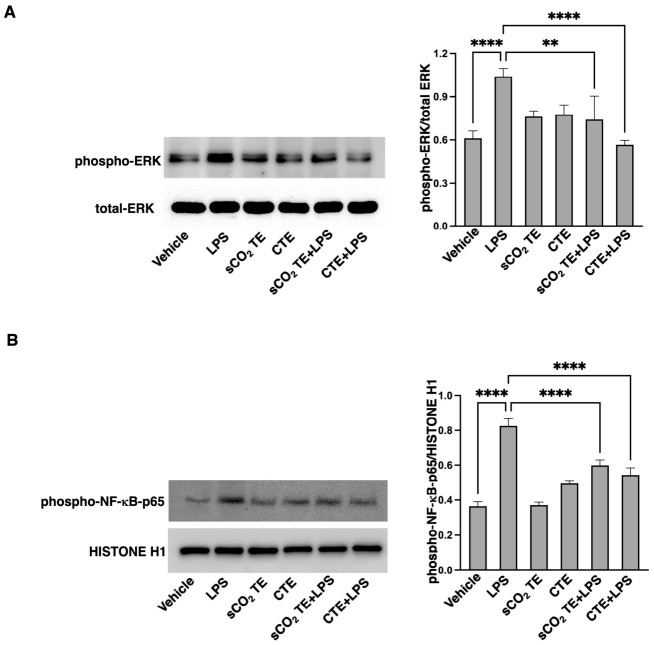
Analysis of MAPK signaling and NF-κB activation after supercritical CO_2_ tomato extract (sCO_2_TE) treatment. Human glioblastoma astrocyte cells U-373 were treated with sCO_2_TE (100 μg/mL), conventional tomato extract (CTE, 100 μg/mL) or LPS (100 ng/mL), and alternatively they were pre-treated with sCO_2_TE (100 μg/mL) and then stimulated with LPS (100 ng/mL). After treatments cells were lysed and analyzed by Western blot. (**A**) Whole cell extracts were analyzed to verify phosphorylated-ERK expression using rabbit anti-phospho-ERK1/2 Ab. (**B**) Nuclear cell extracts were analyzed to verify phosphorylated-NF-kB-p65 expression using rabbit anti-phospho-NF-kB-p65 Ab. As a control, for loading and purity of preparation, membrane was stripped and reprobed with polyclonal anti-HISTONE H1 Ab. Densitometric phospho-ERK/total ERK and phospho-NF-kB-p65/HISTONE H1 ratios are shown in the right panels. Results represent the mean ± SD from 3 independent experiments. Statistical analysis indicates: ** *p* < 0.01; **** *p* < 0.0001.

We analyzed, in U-373 cells, the effect of pre-treatment with tomato extracts on two key molecules involved in the pro-inflammatory signaling activation pathway: mitogen-activated protein kinase (MAPK) ERK1/2 and the p65 subunit of nuclear factor-κB (NF-κB-p65). As expected, LPS stimulation of U-373 cells induced a significant increase in phospho-ERK1/2 (Figure 8A) and phospho-NF-kB-p65 levels (Figure 8B), compared to vehicle. In U-373 cells, pre-treated with tomato extracts and stimulated with LPS, we observed a significant modulation of the pro-inflammatory signaling pathway. In fact, the levels of phospho-ERK1/2 and phospho-NF-kB-p65 appeared comparable to the vehicle sample (Figure 8).

These results indicate that sCO_2_TE, more than CTE, may modulate the intracellular signaling cascade triggered by LPS, thus exerting an anti-inflammatory effect. Furthermore, these findings confirm how the use of supercritical CO_2_ might be a more effective extraction method.

## 4. Discussion

In this study, we investigated the antioxidant and anti-inflammatory properties of TE obtained by sCO_2_ extraction compared to the extract prepared by a conventional extraction method. To this aim, we used the human glioblastoma astrocytoma cell line U-373, which represents a well-established in vitro model for studying the interplay between neuroinflammation and oxidative stress in the context of neurodegenerative disorders [36,37].

Our results demonstrate that both sCO_2_TE and CTE significantly counteract oxidative stress induced by sodium arsenite, as evidenced by the reduction in intracellular ROS levels and lipid peroxidation. These findings are consistent with the well-established antioxidant properties of tomato-derived compounds, including carotenoids such as lycopene, which have been shown to modulate redox homeostasis and protect cellular membranes from oxidative damage.

Cells are constantly exposed to both internal and external sources of ROS and other oxidants, which can exert either protective or injurious effects [38]. At physiological concentrations, ROS are active as signaling molecules in numerous biological processes; however, excessive ROS production leads to oxidative stress, a condition strongly linked to the onset and progression of multiple human pathologies, including neurodegenerative and neuropsychiatric disorders, cardiovascular diseases, diabetes and cancer [1].

In order to evaluate the biological efficacy of tomato-derived bioactives obtained by the technologically advanced and eco-sustainable sCO_2_ extraction method, the study was employed to assess ROS generation, lipid peroxidation and the modulation of key signaling pathways, as well as antioxidant and pro-inflammatory molecules.

Preliminarily, our results demonstrate that neither extract showed intrinsic cytotoxicity nor proapoptotic activity. In our study, we used, as a pro-oxidant agent, the prototypical metalloid AsNaO_2_, commonly employed to reproduce and investigate oxidative stress in cellular models as well as the molecular pathways involved in redox imbalance [29,39]. Interestingly, sCO_2_TE, similarly to CTE, exerts potent cytoprotective effects against oxidative stress in human U-373 cells, leading to an evident reduction in intracellular ROS generation and lipid peroxidation when cells are subjected to AsNaO_2_. Lipid peroxidation represents a critical aspect of oxidative stress, especially in neuronal cells, where membrane integrity is essential for proper function. The ability of both extracts to significantly reduce lipid peroxidation further highlights their protective role at the cellular level and reinforces their potential relevance in neuroprotective strategies.

To counteract the injurious effects of oxidative stress, cells have evolved complex antioxidant defense systems, including the transcription factor NRF2. This is a key regulator of the antioxidant response and cellular protection [7]. Following the production of ROS, NRF2 is phosphorylated and translocates into the nucleus, where it forms heterodimers with small Maf (sMaf) proteins. The phospho-NRF2–sMaf complex then binds to specific promoter sequences in the DNA, known as Antioxidant Response Elements (AREs), activating the transcription of several cytoprotective and antioxidant genes, including SOD and GPX. In addition, NRF2 may interact with other signaling pathways, such as NF-κB, establishing a balance between antioxidant and inflammatory responses [8].

Mechanistically, our results show that sCO_2_TE as well as CTE, activates the NRF2 pathway and, in combination with AsNaO_2_, upregulates downstream antioxidant defenses, including SOD1 and GPX1, thereby enhancing the cellular redox buffering capacity. In addition, pre-treatment with the sCO_2_TE or CTE reduces the LPS-induced phosphorylation of ERK1/2 and NF-κB p65, indicating a consistent anti-inflammatory modulation. These dual antioxidant and anti-inflammatory actions highlight the biological relevance of tomato bioactives in maintaining redox homeostasis under stress conditions. Overall, the effect of sCO_2_TE was very similar, and in some cases higher, to that obtained by conventional extraction method, used as a control.

Our findings indicate an optimal performance of the sCO_2_TE. From a technological perspective, this finding is particularly relevant. Unlike conventional extraction methods that rely on organic solvents, supercritical CO_2_ extraction is a green technology that allows the recovery of bioactive compounds under mild conditions, minimizing degradation and avoiding solvent residues. The preservation of biological activity observed in this study supports the use of this method for the production of high-quality nutraceutical ingredients, since it maintains comparable bioactivity with conventional extraction, offering sustainability advantages [24,40]. These results support the use of supercritical CO_2_ as a sustainable and efficient extraction technology for the development of nutraceutical formulations aimed at mitigating oxidative stress and neuroinflammation.

## Figures and Tables

**Table 1:** Primers for real-time PCR amplification.

Gene	Forward Primer (5′–3′)	Reverse Primer (5′–3′)
*hGAPDH*	ACAGTCAGCCGCATCTTC	GCCCAATACGACCAAATCC
*hSOD1*	GGTCCTCACTTTAATCCTCT	CCAACATGCCTCTCTTCATC
*hNRF2*	TTCAGCCAGCCCAGCACATC	CGTAGCCGAAGAAACCTCAT
*hGPX*	CCAGTTTGGGCATCAGGAGAA	CGAAGAGCATGAAGTTGGGCT

**Table 2:** Characterization of bioactives of sCO_2_TE.

		sCO_2_TE Components (mg gr^−1^ Extract)
Carotenoid	Lycopene	1.34 ± 0.03
Tocopherol	γ-tocopherol + α-tocopherol	5.44 ± 0.11
Phytosterol	Cholesterol	0.28 ± 0.07
	Campesterol	1.21 ± 0.05
	Stigmasterol	2.18 ± 0.02
	β-Sitosterol	2 ± 0.16
	26-Nor-5-cholesten-3-β-ol-25-one	0.16 ± 0.04
	Cycloartenol	0.9 ± 0.07
	lanost-8-en-3-ol	0.27 ± 0.04
	Total	7

sCO_2_TE: supercritical CO_2_ tomato extract.

## Data Availability

The datasets used and analyzed during the current study are available from the corresponding author upon reasonable request.

## Supplementary Materials

The following supporting information can be downloaded at: https://www.mdpi.com/article/10.3390/nu18091464/s1, Figure S1: Preliminary analysis on effect of sodium arsenite (AsNaO_2_), supercritical CO_2_ tomato extract (sCO_2_TE) and conventional tomato extract (CTE) on cell viability.
