# Supplementary material for: Supercritical CO2-Derived Tomato Extract Activates Signaling Pathways to Reduce Oxidative Stress and Inflammation in Astrocyte Cells
Source: Nutrients. 2026 May 3;18(9):1464. doi: 10.3390/nu18091464 (PMC13165377; doi:10.3390/nu18091464)
Supplement: Supplementary file 1 [file nutrients-18-01464-s001.zip › Figure S1.pdf]

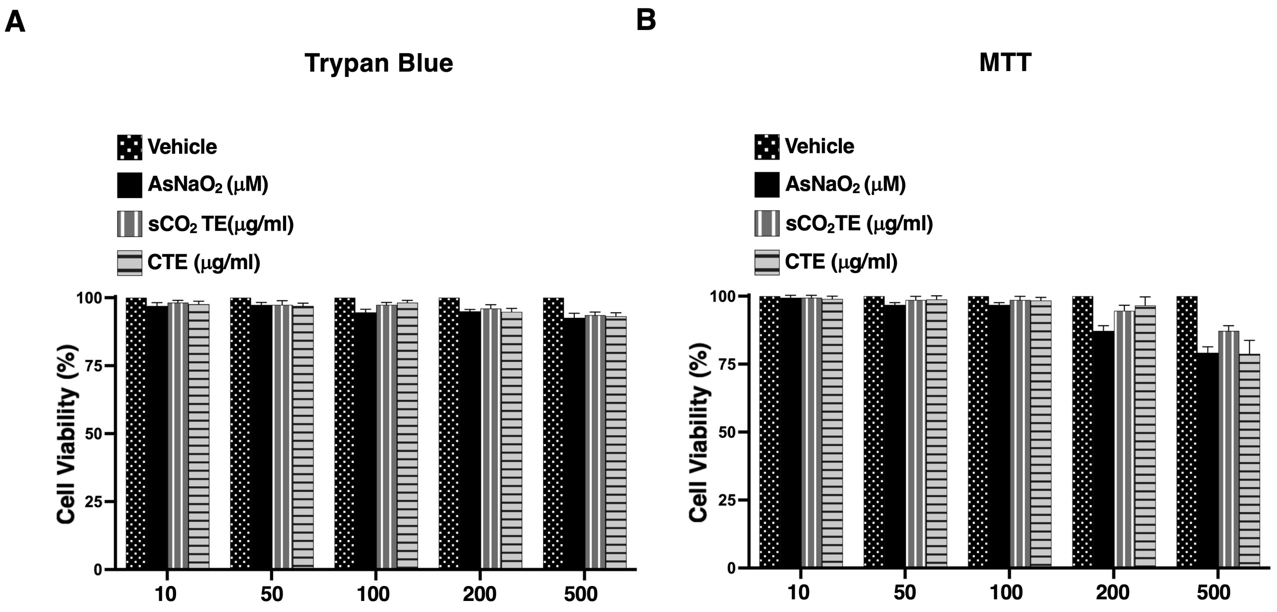

Figure S1. Preliminary analysis on effect of sodium arsenite (AsNaO2), supercritical CO2 tomato extract (sCO2TE) and conventional tomato extract (CTE) on cell viability.
